# The Molecular Characteristics of Non-Clear Cell Renal Cell Carcinoma: What’s the Story Morning Glory?

**DOI:** 10.3390/ijms22126237

**Published:** 2021-06-09

**Authors:** Andrea Marchetti, Matteo Rosellini, Veronica Mollica, Alessandro Rizzo, Elisa Tassinari, Giacomo Nuvola, Alessia Cimadamore, Matteo Santoni, Michelangelo Fiorentino, Rodolfo Montironi, Francesco Massari

**Affiliations:** 1 Medical Oncology, IRCCS Azienda Ospedaliero-Universitaria di Bologna, Via Albertoni—15, 40138 Bologna, Italy; andrea.marchetti12@studio.unibo.it (A.M.); matteorosellini92@gmail.com (M.R.); veronica.mollica7@gmail.com (V.M.); rizzo.alessandro179@gmail.com (A.R.); elisa.tassinari95@gmail.com (E.T.); giacomo.nuvola87@gmail.com (G.N.); 2 Section of Pathological Anatomy, School of Medicine, Polytechnic University of the Marche Region, United Hospitals, 60126 Ancona, Italy; a.cimadamore@staff.univpm.it (A.C.); r.montironi@staff.univpm.it (R.M.); 3 Oncology Unit, Macerata Hospital, 62100 Macerata, Italy; mattymo@alice.it; 4 Department of Specialistic Diagnostic and Experimental Medicine, University of Bologna, Via Massarenti 9, 40138 Bologna, Italy; michelangelo.fiorentino@unibo.it

**Keywords:** non-clear cell renal cell carcinoma, papillary RCC, chromophobe RCC, TKI, immunotherapy, molecular targets, pathways, VEGFR, mTOR, MET

## Abstract

Non-clear cell renal cell carcinomas are a miscellaneous group of tumors that include different histological subtypes, each one characterized by peculiarity in terms of genetic alteration, clinical behavior, prognosis, and treatment response. Because of their low incidence and poor enrollment in clinical trials, alongside their heterogeneity, additional efforts are required to better unveil the pathogenetic mechanisms and, consequently, to improve the treatment algorithm. Nowadays, tyrosine kinase inhibitors, mTOR and MET inhibitors, and even cisplatin-based chemotherapy and immunotherapy are potential weapons that are still under evaluation in this setting. Various biomarkers have been evaluated for detecting progression and monitoring renal cell carcinoma, but more studies are necessary to improve this field. In this review, we provide an overview on the molecular characteristics of this group of tumors and the recently published trials, giving an insight into what might become the future therapeutic standard in this complex world of non-clear cell kidney cancers.

## 1. Introduction

Renal cell carcinoma (RCC) is the 7th and the 10th most common cancer in male and female population respectively, with a worldwide estimate for 2020 of more than 431,000 new cases and more than 179,000 deaths [1]. The incidence of this cancer is greater from the sixth to eighth decades and usually they are diagnosticated during exams performed for other reasons [2]. More than 65% of all RCC are localized at the diagnosis and in this setting 90% of patients survive at least 5 years [3]. On the other hand, the prognosis is poorer in metastatic patients and the mortality rate per year is about 4/100,000 people in the Western world. [4].

Generally, kidney cancer can be divided in two main histological groups: clear cell renal cell carcinoma (ccRCC), representing about 75–80% of all cases of RCC, and non-clear cell renal cell carcinoma (nccRCC), that includes several histologic subtypes differing in terms of cellular morphology, molecular alterations and gene expression, prognosis, and response to therapy. Among nccRCC, the most frequent histologic entities are chromophobe (chRCC) and papillary renal cell carcinoma (pRCC). The latter can be distinguished into type 1 pRCC (basophilic) and type 2 pRCC (eosinophilic). Nonetheless, there are many less common subtypes considered part of the nccRCC group: medullary RCC, collecting duct RCC, oncocytoma, translocation RCC, tubulocystic RCC, and hereditary leiomyomatosis and RCC (HLRCC)-associated RCC [5,6]. About 5% of RCC remain unclassified due to their histological features that do not fit into any of the well-recognized subtypes [6]. Even though most nccRCCs tumors are sporadic, approximately 5% may present a genetic predisposition [7]. Hereditary renal cancers are more often multiple and bilateral and, usually, occur at an earlier age than the non-familial renal tumors [8].

Nowadays, our knowledge about the sarcomatoid and/or rhabdoid dedifferentiation of RCC (S/R RCC) patients is typically associated with poor prognosis. Even though the treatment scenario for metastatic ccRCC has significantly evolved over the past decade, with multiple targeted therapies and immune checkpoint inhibitors (ICI)-based regimens approved, very little progress has been made in the therapeutic management of advanced nccRCC [9,10]. As a matter of fact, patients with metastatic nccRCC have usually not been included in phase III trials, due to the low incidence of these tumors along with their clinical and molecular heterogeneity. This lack of available data has led physicians to treat nccRCC patients with a limited number of therapies, even adopting the same algorithm used in ccRCC.

**Figure 1:**
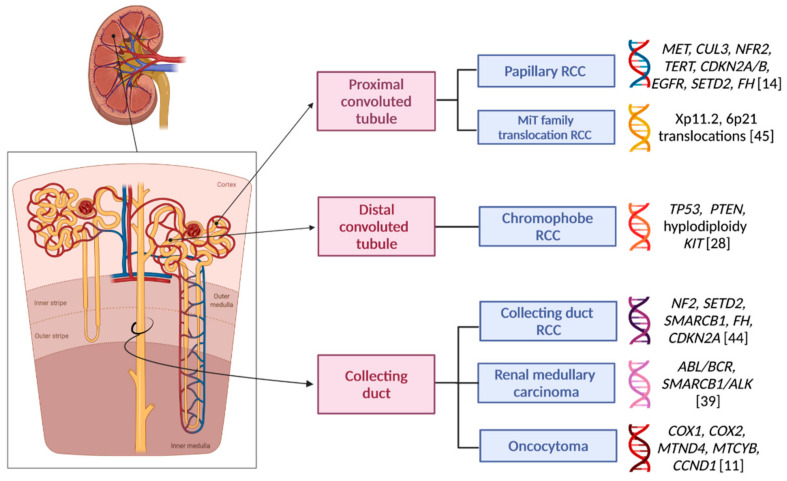
The papillary renal cell carcinoma (pRCC) has been shown to originate from the proximal convoluted tubule’s epithelium, along with the MiT family translocation RCCs. Chromophobe RCC originates from intercalated cells in the distal convoluted tubule, while collecting duct RCC, oncocytoma, and renal medullary carcinoma (RMC) appear to originate from distal collecting duct principal or calyceal cells [6]. In this figure the most common genetic abnormalities are suggested for each of these non-clear cell histotypes [11]. Created by BioRender.com (accessed on 8 June 2021).

In the last few years, an extensive molecular description of the key pathogenetic alterations in the major forms of tumors, among which RCC is noteworthy, has been performed by the Cancer Genome Atlas (TCGA), a collaboration between the National Cancer Institute and the National Human Genome Research Institute [11]. Nowadays the increased knowledge of these molecular findings grants a novel integration of histopathological and genomic aspects, which progressively paves the way for a personalization of RCC clinical management. Focusing on nccRCC, the recent genome sequencing studies have highlighted how important it is to consider the many subtypes included in this group as standalone entities, since each of these is characterized by a specific spectrum of molecular altered pathways differing from the tumor’s morphology, immune-histochemical features, but also response to systemic treatments, clinical behavior, and prognosis (Figure 1) [12]. These pivotal differences could be exploited to improve the therapeutic management of nccRCC.

**Figure 2:**
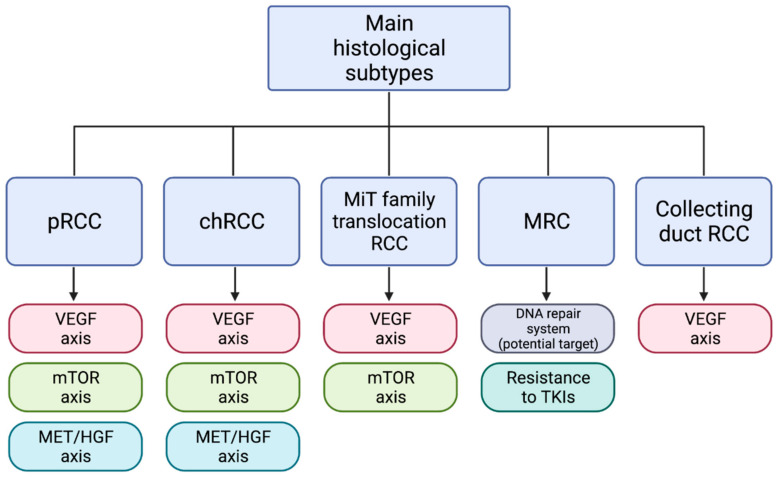
Workflow diagram of the molecular targets of the main histological subtypes of nccRCC. Abbreviations: pRCC = papillary renal cell carcinoma; chRCC = chromophobe RCC; MRC = medullary renal cell carcinoma; VEGF = vascular endothelial growth factor; mTOR = mammalian target of rapamycin; MET = mesenchymal epithelial transition or hepatocyte growth factor receptor; HGF = hepatocyte growth factor; TKIs = tyrosine kinase inhibitors. Created by BioRender.com (accessed on 8 June 2021).

The aim of this paper is to describe the most important signaling pathways involved in the nccRCC oncogenesis, highlighting the newly described molecular characteristics of each histotype, and mostly paying attention to potential targets for current and future therapeutic strategies (Figure 2). We also discuss the molecular characterization of RCC with sarcomatoid dedifferentiation and their management in the light of recent published trials. In addition, we provide an overview of the most significant ongoing and recently published trials (testing immunotherapies or targeted therapies) that involved patients with nccRCC. We performed a research on PubMed/Medline, Cochrane Library, and Scopus using the keywords “non-clear cell renal cell carcinom", “advanced nccRCC”, “metastatic nccRCC”, and “molecular characterization” or “genomic profiling” or “molecular targets”. We consulted case reports, reviews, and original articles, from 1996 to the current year.

## 2. Main Histological Subtypes

### 2.1. Papillary RCC

The most frequent subtype of nccRCC is papillary RCC (pRCC), which is also the second most common carcinoma of the renal tubules after ccRCC, accounting for 10–15% of all kidney cancers. In more detail, pRCC is a heterogeneous disease that involves indolent malignancies with a multifocal presentation as well as solitary tumors with an aggressive phenotype. As for the histological aspect, pRCC is constituted by malignant cells histologically arranged in a papillary pattern, that are derived from proximal tubule epithelium. The papillae are supported by a fibrous-vascular axis, infiltrated by foamy macrophages, and characterized by cholesterinic necrosis and psammoma bodies. Frequent hemorrhagic or necrotic foci and cystic degeneration are described. Of note, a poor prognosis may be correlated with the sarcomatoid dedifferentiation of the tumor along with the coexistence of clear cells [13]. The entity of pRCC is classically subdivided in two main subtypes, type 1 and type 2 pRCC, based on their histopathological appearance. Furthermore, type 1 and type 2 pRCC are shown to differ from each other due to their clinical and also biological features [5,14]. Type 1 pRCC is typically associated with better prognosis and a more indolent behavior: it has a slow growth rate along with a low tendency to metastasize. From a microscopic point of view, it is characterized by regular layers of basophilic cells with large round nuclei and poor cytoplasm, as well as a poor vascular bed. On the other side, aggregates of eosinophilic cells organized to form papillae are found in type 2 pRCC, highlighting more aggressive features, such as polymorphic irregular nuclei with nucleoli, infiltrating foamy macrophages and psammoma bodies [15]. Moreover, type 2 pRCC is characterized by a higher pathological grade and worse tumor stage at the moment of the diagnosis than type 1 pRCC, confirming the worst course of this disease. As a matter of fact, type 2 pRCC is associated with poorer survival outcomes [16,17]. According to the 2016 WHO classification, mixed histologies have been recently accepted as part of the pRCC group, including the so-called oncocytic-papillary RCC or the clear cell papillary RCC (ccpRCC) [5,18]. The first studies on the pRCC genomic profile were conducted in the late 1990s, focusing on patients affected by hereditary pRCC (HPRCC). Researchers identified activating mutations of *MET* proto-oncogene as the main pathogenetic mechanism in the development of this hereditary cancer syndrome [19]. Further genomic profiling studies on somatic pRCC patients showed how commonly *MET* mutations may be discovered in type 1 pRCC cases [14]. Notably, sporadic type 1 pRCC is associated with genetic abnormalities, such as gains or trisomy of chromosomes 7 (whose long arm contains *MET* proto-oncogene), 17 and less frequently 2, 3, 12, 16, and 20. On the other hand, type 2 pRCC has been recently shown to be a heterogeneous group, which may be divided into three separate subtypes, according to their molecular differences along with patients’ survival outcomes. Mutations of *SETD2*, *NF2, CUL3, TERT* promoter, as well as *CDKN2A* silencing, increased expression of the NRF2-antioxidant pathway and gains of chromosomes 7, 12, 16, and 17 are described in sporadic type 2 pRCC patients [20,21]. Negative prognostic factors observed in type 2 pRCC are represented by CpG island methylator phenotype and 9p loss [14]. Genetic alterations of the fumarate hydratase gene (*HF*) have been frequently displayed in a rare hereditary form of type 2 pRCC, described among patients affected by hereditary leiomyomatosis RCC (HLRCC) [12,22]. The above-mentioned molecular differences between type 1 and type 2 pRCC have been reported in the Cancer Genome Atlas (TCGA) profiling, which largely reflects localized disease. As for advanced disease, other molecular differences have been described between type 1 pRCC (*MET* 33%, *TERT* 30%, *CDKN2A/B* 13%, and *EGFR* 8%) and type 2 pRCC (*CDKN2A/B* 18%, *TERT* 18%, *NF2* 13%, *FH* 13%, and *MET* 7%) in further studies. These differences are extremely noteworthy due to their future use as targets for specific therapies for the metastatic setting management [23].

### 2.2. Chromophobe RCC

Chromophobe renal cell carcinoma (chRCC) is the third most common subtype of RCC, representing about 5% of all the cases and originates from the intercalated cells of the collecting duct [24].

This neoplasia is characterized by two main varieties of cells: pale and eosinophilic cells. Pale cells are large with rich cytoplasm and plant-like membrana. The eosinophilic ones are smaller and present granular eosinophilic cytoplasm. The eosinophilic variant is predominantly composed of these latter types of cells [25]. A Furham or WHO/ISUP nuclear grade cannot be assigned due to the abundant nuclear atypia [26]. Usually, this tumor affects young patients, mostly female, and it is mainly diagnosed at an early stage [27]. A peculiar aspect of chRCC is its aneuploidy, given by substantial chromosomal loss, while somatic mutations are less frequent than in ccRCC. Among these, *TP53* and *PTEN* are the most common alterations (*PTEN* is associated with poor prognosis), even if it is possible to detect mutations also in mTOR, *NRAS*, and *TSC1* and *2* [28,29]. Several rare genetic syndromes are associated with chRCC: Birt–Hogg–Dubé syndrome, BAP1 tumor predisposition syndrome, tuberous sclerosis, Cowden syndrome, and hereditary paraganglioma/pheochromocytoma syndrome (all autosomal dominant diseases) [30]. Generally, chromophobe RCC has a better prognosis if compared to ccRCC (with greater than 90% 10-year survival) but the presence of sarcomatoid differentiation provides a more aggressive behavior and a worse outcome [31].

### 2.3. RCC with Sarcomatoid/Rhabdoid Features

Sarcomatoid and rhabdoid renal cell carcinoma (S/R RCC) represents an aggressive form of dedifferentiation of RCC, coexisting with other variants in approximately 10–15% of patients, whichever is the predominant subtype of kidney cancer. Sarcomatoid and rhabdoid features may occur in the same neoplasia or independently from each other, and it represents the progressive evolution from a differentiated to an undifferentiated RCC. As a consequence, S/R RCC carries a poor prognosis, being characterized by an aggressive behavior; as a matter of fact, most patients with S/R RCC are metastatic at the diagnosis and witness a rapid clinical progression [32,33]. Both the sarcomatoid and epithelial components of the neoplasia share a common progenitor cell, but the clonal evolution during the oncogenesis leads to several genetic abnormalities in cells that are going to take part of the sarcomatoid component, triggering the so-called epithelial-to-mesenchymal transition (EMT) and hesitating in intratumoral genetic heterogeneity [34]. An expanded clinical and molecular integrated characterization of S/R RCC, built on data from clinical trials and real-world cohorts, has been recently carried out by Bakouny and colleagues, shedding light on the molecular drivers of aggressivity and responsiveness to therapies of these so far little known histotypes [35]. First of all, S/R RCCs are characterized by the enrichment of *MYC*-regulated genes, along with multiple molecular pathways involved in cell cycle regulation and tumor’s invasiveness. The upregulation of these *MYC*-related genes correlates with the poor quoad vitam prognosis of these histotypes, thus unveiling its pathogenetic key role as molecular driver of aggressivity of S/R RCCs [35]. According to prior studies, several mutations regarding the Hippo pathway (including *NF2* gene’s ones) have been marked in sarcomatoid RCC, while *BAP1* abnormalities have been shown to relate to sarcomatoid as well as rhabdoid RCC tumors. *CDKN2A/B* deletions have been reported more frequently in S RCC tumors, even though they are also present in R RCC and non-S/R RCC tumors. Moreover, we may keep in mind, the depletion of S/R RCC molecular markers in *KDM5C* mutations and the enrichment in *EZH2* amplifications, too [36,37,38]. The overexpression of aurora kinase A with an increased mTOR activity has been described in sarcomatoid cells sampled from eight nephrectomized patients, with the help of RNA sequencing. Nonetheless, mTOR inhibitors seem not to be so effective in S/R RCC treatment [39]. Furthermore, the current characterization highlights the increased PD-L1 expression in S/R RCC malignant cells, along with an enriched CD8+ T cell intratumoral infiltration. Notably, S/R RCC tumors are shown to exhibit an immuno-inflamed phenotype, explaining consequently their noticeable responsiveness to ICIs. S/R RCC cells display an overexpression of all 8 “Hallmark” immune gene sets (including IL6-JAK-STAT3 signaling and IF gamma response). Moreover, an immune responsive microenvironment is significantly expressed in S/R RCC tumors, including elements such as M1 macrophages, Th1 T cell subsets, or activated NK cells [35]. Lastly, mutations in specific tumor suppressor genes (in particular *TP53*, but also *ARID1A*) are described as very common findings in sarcomatoid and rhabdoid components of S/R RCCs [40].

### 2.4. Collecting Duct RCC

Collecting duct RCC (cdRCC; also known as Bellini duct carcinoma) originates from the principal cells of distal collecting ducts, accounting for less than 1% of all RCC. It has a very poor prognosis and about 1 patient of 3 presents metastasis at the diagnosis. This rare subtype of RCC is composed of two main histological features, a desmoplastic stroma and a glandular part with an atypical epithelium [41]. It has been observed a morphologic and a cytogenetic similarity with urothelian cell carcinoma, also sharing a close clinical behavior [42]. For these reasons, chemotherapy regimens also used in urothelial carcinoma may be appropriate, although the prognosis still remains poor [43]. A NGS of prespecified genes panel performed on 17 patients with cdRCC highlighted genomic alterations in *SETD2*, *CDKN2A*, *SMARCB1,* and *NF2* (the latter has been revealed in one third of the cohort) [44].

### 2.5. MiT Family Translocation RCC

MiT family translocation RCC is a rare and aggressive variety of RCC arising in the pediatric population and in young adults. This group of aggressive tumors are characterized by various translocations, most of them involve the transcription factor E3 (*TFE3*) that is located on chromosome X (Xp11.2) [45]. Tumors associated with Xp11 and t(6;11) translocations are the most frequent and morphologically described tumors of this family. Xp11 translocation and, in general, MiT family translocation RCC that develop in adult age could have an aggressive behavior and spread early in lymph nodes [46].

Translocation RCC presents a papillary and/or alveolar pattern, with large eosinophilic and/or clear cells and the pathological diagnosis is supported by the nuclear immunoreactivity of *TFE3* [47]. Moreover, TFE3-positive tumors are more likely to be diagnosed at an advanced stage and a more aggressive behavior is noticed when it displays in adults [48,49].

### 2.6. Renal Medullary Carcinoma

Renal medullary carcinoma (RMC) is a rare and extremely aggressive variant of renal cell carcinoma and mainly affects African young male with sickle cell trait. Less than 5% of patients survive more than 3 years [50]. RMC is treated with platinum-based chemotherapy due to its intrinsic resistance to all the targeted therapy used in the other RCCs. New data coming from recent work suggest the potential role of agents targeting replication stress pathways (for example PARP inhibitors) in the treatment of this rare subtype, but further investigations are required [51]. Transcriptomic analysis of RMC samples showed similar gene expression features between RMC and chRCC. One of the most frequent alterations evidenced in RMC is in *SMARCB1* gene [39].

In Table 1 are reported the non-clear cell histological subtypes of RCC with their features.

## 3. Molecular Targets

### 3.1. VEGF Axis Pathway

The role of angiogenesis in the growth of organs and tumors is widely recognized. Angiogenesis is an intricate process strictly regulated via a complex balance between proangiogenic and antiangiogenic factors, whose synthesis and excretion may depend on the tumor itself as well as the neighboring extracellular matrix cells in the malignant microenvironment [52]. Chemokines, a superfamily of structurally homologous heparin-binding proteins, participate in this complex mechanism due to their power to induce and even inhibit angiogenesis [53].

With regards to malignant tumors, the above-described tight equilibrium is titled in favor of angiogenesis. In more detail, the abnormal cancer-related angiogenesis appears to be frequently related to tumor hypoxia, caused by an uncontrolled malignant proliferation contemporary to a poor expansion of the vascular bed [54]. Among the pro-angiogenic factors, the vascular endothelial growth factor (VEGF) is the pivotal heparin-binding polypeptide that modulates angiogenesis by interacting with tyrosine kinase VEGF receptors, VEGFR1 (also known as FLT1) and VEGFR2 (also indicated as KDR or FLK1) [55]. The synthesis of VEGF is induced by hypoxia, and the VEGF signaling pathway appears to be upregulated in several human cancers, with a remarkable and well-known implication in RCC tumorigenesis. Notably, the biallelic inactivation of the Von Hippel-Lindau (*VHL*) tumor suppressor gene occurs in the vast majority of RCC (particularly in the clear cell histotype), leading to the accumulation of hypoxia-inducible factors and overexpression of several genes, including those for VEGF [56]. By the way, different RCC histotypes appear to be related to different expressions of VEGF, VEGFR1, and VEGFR2 mRNA levels. The expression patterns of VEGF and its tyrosine kinase receptors were shown to be higher in ccRCC than in pRCC in a 2006 Swedish study, pointing out that different pathways are likely involved in regulating angiogenesis in the different RCC histotypes [57]. As proof of this, the initial oncogenic event for nccRCC seems not to be VHL-driven, explaining the poor rate of *VHL* mutations among non-clear cell histologies [58]. The importance of this finding is to be found in the resulting therapeutic management of nccRCC. Patients with metastatic nccRCC are characterized by a worse response to VEGF/VEGFR-targeted therapies, as well as a shorter OS, if compared to ccRCC patients [4]. Of interest, RCC patients with wild type *VHL* along with *TP53* and *FLT1* mutations were shown to be related with shorter progression-free survival (PFS) in response to VEGF-targeted treatments, compared to RCC patients with genetic abnormalities of the *VHL* gene. As described above, these genetic profiles distinguish non-clear cell histotypes, remarking the worse response to VEGF-targeted therapies of nccRCCs [59]. A still-today pivotal VEGFR-targeted agent in metastatic ccRCC treatment as sunitinib did not display the same expected efficacy when investigated in the non-clear cell setting. The prospective phase II trials ASPEN and ESPN compared the administration of sunitinib with mTOR inhibitor everolimus in metastatic nccRCC patients not previously treated with TKIs. Whereas the ASPEN trial reported a better PFS in the sunitinib group compared to patients treated with everolimus (hazard ratio (HR) 1.41, 80% confidence interval (CI): 1.03–1.92, *p* = 0.16) along with a comparable mOS (HR 1.12, 95% CI: 0.7–2.1) [60], no survival benefits were shown to be related to the administration of sunitinib if compared to everolimus, in the ESPN study (HR for PFS 1.16, 95% CI: 0.67–2.01) [61].

Similar PFS rates for sunitinib and everolimus (HR 1.5, 95% CI: 0.9–2.8) with regards to non-clear cell histotypes were shown in subgroup analysis of the 2014 phase II RECORD-3 trial, which was designed to compare first-line everolimus followed by sunitinib at progression with the standard sequence of first-line sunitinib followed by everolimus in metastatic RCC [62]. According to further genomic studies on RECORD-3 enrolled patients, *KDM5C* mutations were related to longer PFS (20.6 months) if compared to *PBRM1* (11 months) and *BAP1* mutations (8.1 months) among patients treated with first-line sunitinib, suggesting a role of these genetic abnormalities in granting VEGFR-targeted therapy sensibility. On the other side, PBMR1 alterations were associated with PFS benefits compared to *BAP1* mutations (12.8 months vs. 4.9 months) in case of first-line treatment with everolimus [63].

However, even though the meta-analysis of ESPN and ASPEN pooled data did not display any significant difference in terms of PFS (HR 1.3, *p* = 0.15), a trend for superiority of sunitinib over everolimus was demonstrated, thus explaining the clinical recommendation preferring sunitinib over mTOR inhibitors [4]. This advantage of sunitinib in terms of survival benefits was recently reached when compared to temsirolimus, in a current phase IIa trial as well [64]. Moreover, the clinical activity of sunitinib in untreated patients with locally advanced or metastatic type 1 and type 2 pRCC was proved in a phase II SUPAP trial, despite being lower than in clear cell histotype (Table 2) [65]. To date there is no evidence supporting the use of the VEGF-aimed monoclonal antibody bevacizumab as monotherapy in nccRCC patients. Nevertheless, the combination of bevacizumab with everolimus appeared to have an encouraging clinical activity when used as a first-line strategy in advanced nccRCC patients, especially in case of papillary histotype as well as unclassified renal cancer with a major papillary component [66]. The biological rationale of this combination strategy is to be found in the mTOR pathway activation after the tumor hypoxia induced by VEGF-targeting treatments [67]. As for other VEFGR-targeted tyrosine kinase inhibitors, a small non-randomized trial supported the efficacy of sorafenib among papillary and chromophobe RCC patients, although it seemed to be limited if compared with sunitinib in the same setting [68]. Most of the data evaluating the efficacy of pazopanib in metastatic nccRCC derived from retrospective studies, pointing out its use as a possible therapeutic option in this setting. Since comparator trials are missing and prospective data are strictly limited, the administration of this latter TKI outside clinical trials is not routinely recommended [69].

### 3.2. mTOR Pathway

The protein kinase mTOR (mechanistic target of rapamycin) controls cell growth and its metabolism and it is usually assembled into several complexes such as mTOR complex 1/2 (mTORC1/2) [70].

Even the mTOR signaling pathway has a relevant role in the development of kidney cancer and it represents an attractive therapeutic target among several subgroups of RCC. The activation of this axis provokes the upregulation of hypoxia-inducible factor 1α (HIF-1α), a transcription factor that regulates the expression of various genes (VEGF among others) involved in adjusting mechanisms to hypoxia such as angiogenesis, apoptosis, or tumor metastasis [71].

Preliminary data about the efficacy of blocking HIF-2α in heavily pretreated ccRCC emerged from a recent phase I study performed by Choueiri, in which the new compound belzutifan (MK-6482) showed a promising anti-tumor activity, with a good safety profile. It would be interesting to test belzutifan in new trials involving patients with nccRCC, to explore its potential role in this setting [72].

In Cowden disease, a hereditary autosomal dominant syndrome associated with germline mutation of *PTEN*, which is an onco-suppressor gene that inhibits the PI3K/Akt/mTOR pathway, making it possible to develop clear cell, papillary, and chromophobe RCC [73]. Therefore, in this context, targeting mTOR represents an effective strategy and the same benefit could be achieved even in other genetic syndromes.

Tuberous sclerosis complex (TSC)-associated kidney cancer is another autosomal dominant syndrome characterized by different renal tumors (for example, angiomyolipoma and its malignant variant epithelioid angiomyolipoma) and caused by alterations in two genes, *TSC1* and *TSC2*, that induce the constitutive activation of the LKB1/AMPK/TSC/mTOR pathway [74]. Positive results of EXIST-2 trial have led to the approbation of the mTOR inhibitor everolimus for the treatment of TSC-associated AML in many countries [75].

As far as autosomal dominant BHD syndrome, the most represented renal tumors are ccRCC, eosinophilic chromophobe RCC, oncocytoma, and hybrid oncocytic. *FLCN* is the tumor suppressor gene linked to the development of this pathology and its homozygous loss induces mTORC1 and 2 activation [76]. This molecular rationale justified the use of the inhibition of mTOR but further studies are needed to determine if this group of therapies provides benefit in patients with BHD-associated renal cell carcinoma [77].

In Xp11 translocation RCC, the mTOR and HIF-1α pathway are normally upregulated, as demonstrated by the increased expression of the downstream molecule phosphorylated S6 [78]. A study published in December 2018 identified TFE3/IRS-1/PI3K/AKT/mTOR as a potential altered pathway in TFE3-tRCC and suggested a potential inhibition of this axis with a dual PI3K/mTOR inhibitor [79].

SDH-RCC (succinate dehydrogenase deficient RCC) and HLRCC (hereditary leiomyomatosis with RCC) reflect the Warburg-effect (preferential anaerobic glycolysis by the tumor’s cells even in presence of oxygen) in renal cell carcinoma. The first-mentioned syndrome is a hereditary disease linked to the predisposition to aggressive type II papillary RCC and it is caused by the mutation of the gene that encodes FH, an enzyme involved in the Krebs cycle. This genetic alteration produces an accumulation of HIF-1α that promotes the anaerobic glycolysis of the Warburg-effect [80]. Moreover, this syndrome is characterized by lower levels of AMPK and upregulation of mTOR pathway, representing a possible therapeutic target for this patient.

On the other hand, mutations of succinate dehydrogenase genes SCHD, SDHB, and SDHC represent the genetic hallmark of SDH-RCC [81]. This alteration induces increased levels of succinate with the subsequent HIF accumulation. In accordance with this biological evidence, targeting mTOR pathways or angiogenesis may reduce glucose uptake by tumor cells, interfering in this way with the proliferation of both these hereditary cancer diseases [82].

As regards the clinical efficacy in inhibiting mTOR in non-clear cell RCC, the phase II trial RAPTOR evaluated everolimus as first-line therapy in 92 treatment-naïve metastatic patients with papillary RCC, showing in the intention-to-treat (ITT) population a median PFS of 4.1 months (95% CI 3.6–5.5) and median OS was 21.4 months (95% CI 15.4–28.4) [83].

The subgroup analysis of the RAD001 Expanded Access Clinical Trial (REACT) demonstrated some benefit for everolimus: a PFS of 2.8 months and an ORR of 50.6%, with 1.3% of PR and with 49.3% of patients that presented stable disease [84].

Another phase II trial revealed a PFS of 13.1 months in patients with chromophobe histotype versus 3.4 months in the other nccRCC subgroups (*p* = 0.084) [85].

Temsirolimus also documented a clinical benefit in patients with nccRCC, especially in the chromophobe histotype, [86] and provided more clinical benefit compared with interferon, regardless of renal tumor histology [87].

### 3.3. MET Pathway

As stated before, papillary RCC are closely linked to activating mutations of the MET proto-oncogene on chromosome 7q31 [19]. *MET* mesenchymal epithelial transition or hepatocyte growth factor receptor) gene encodes for the tyrosine kinase receptor of the hepatocyte growth factor (HGF), which is normally expressed on epithelial and endothelial cells plasma membrane. When it is bound by the inactive serine-protease analog HGF, this tyrosine kinase receptor causes the activation of RAS/MAPK and PI3K/AKT pathways, leading to the expression of multiple genes involved in several physiological functions, such as embryonic organogenesis or adult tissue regeneration, as well as pathological phenomena including tumor growth, malignant cell infiltration, and metastasis [88]. *MET* is shown to share signaling intermediates with the VEGFR axis. ERK, MAPK, AKT, and FAK may be activated by the MET/HGF axis as well as by VEGFR. Moreover, VEGFa expression and angiogenesis may be induced by the MET signaling pathway through SRC homology 2 domain-containing proteins (SHCs). The overexpression of hypoxia-induced factors HIF-1α and HIF-1β results in MET expression as well [89]. In addition, the upregulation of the MET/HGF axis is linked to the acquired resistance to previous VEGFR-targeted therapies displayed in metastatic RCC patients [90].

Whereas activating mutations in MET proto-oncogene are commonly described in the germline of HPRCC patients [19], somatic *MET* alterations have a driver role in about 13–20% of sporadic type 1 pRCCs [91]. As a consequence, the MET/HGF axis must be considered another pivotal therapeutic target in RCC, especially with regards to papillary nccRCCs. Among MET-directed inhibitory agents, we may keep in mind foretinib, tivantinib, crizotinib, but mostly cabozantinib and savolitinib.

The final results of the SWOG-1500 phase II trial (also known as PAPMET) have been recently presented by Pal and colleagues at the American Society of Clinical Oncology Genito-urinary (ASCO GU) Cancers Symposium 2021 [92]. In this study, the clinical activity of the current standard-of-care sunitinib was compared to multi-kinase inhibitors cabozantinib, crizotinib, and savolitinib in 152 patients with metastatic pRCC (up to one prior systemic therapy was permitted). Cabozantinib was shown to reach the primary endpoint with a gain in terms of PFS (9.0 months) in comparison to sunitinib (5.6 months), regardless of the MET status (HR 0.60, 95% CI 0.37–0.97; one-sided *p* = 0.019). On the other side, the enrollment in the other two arms of savolitinib and crizotinib was stopped in advance on the basis of a preplanned futility analysis. These findings suggested a key role of both VEGF- and MET-signaling pathways in pRCC’s oncogenesis, thereby explaining its higher responsiveness to a dual VEGF/MET inhibitor such as cabozantinib than to more selective MET inhibitors as crizotinib or savolitinib [92]. Therefore, cabozantinib may be considered as a novel practice changing strategy for metastatic pRCC patients (Table 2). Taking into account the noteworthy results of the recent phase III CheckMate 9ER trial in the metastatic clear cell setting, it would be of interest assessing the clinical activity of cabozantinib in combination with an ICI (such as the PD-L1 inhibitor atezolizumab in the ongoing COSMIC-021 and CONTACT-03 studies) for an even more effective management of pRCCs [93,94]. As for savolitinib, in a 2017 phase II study this selective MET-inhibitor unveiled promising activity in metastatic pRCC with MET-driven mutations (i.e., gains in chromosome 7, MET amplifications, HGF alterations, and so on) if compared to the MET-independent disease (median PFS was 6.2 months vs. 1.4 months, respectively; HR 0.33, 95% CI: 0.20–0.52; *p* < 0.001) [95]. Moreover, the 2020 phase III SAVOIR trial compared the administration of savolitinib to sunitinib in patients with advanced MET-driven pRCC (Table 2) [96]. Although this study did not meet its primary endpoint of PFS (HR 0.71; 95% CI, 0.37–1.36; *p* = 0.31) probably due to its design as well as the low accrual of patients, savolitinib was shown to achieve a significantly better response rate than sunitinib (ORR 27% vs. 7%), along with a better safety profile [97]. Likewise, crizotinib might ensure more benefits if used in patients with metastatic pRCC characterized by MET-driven mutations. As a matter of fact, the CREATE trial highlighted the correlation between the administration of crizotinib and the higher response rate and long-lasting disease control in metastatic type 1 pRCC patients with MET-driven mutations, if compared to patients with MET-independent disease (Table 2) [98]. Further comparative studies designed with a genomically driven approach would be needed to better explore the activity of savolitinib and crizotinib in this setting.

Foretinib (a multi-kinase inhibitor targeting MET, VEGFR-2, RON, and AXL) was one of the first MET-targeted agents investigated, and it showed a promising clinical activity with a manageable safety profile in a multicenter phase II trial of patients with sporadic and hereditary pRCC. The preplanned subgroup analysis unveiled a higher response rate obtained with foretinib in patients with germline MET-driven mutations than in patients without these genetic alterations [99]. Conversely, the selective MET-inhibitor tivantinib, either alone or in combination with EGFR-inhibitor erlotinib, displayed no clinical activity among patients with advanced pRCC (Table 2) [100].

The involvement of the MET/HGF axis is also related to non-papillary nccRCC’s oncogenesis. For example, the aberrant ASPL-TFE3 fusion protein in MiT family translocation RCC seems to induce the overexpression of *MET* proto-oncogene by binding its promoter region, leading to MET autophosphorylation and activation of downstream signaling in the presence of HGF [101]. Nevertheless, available data regarding the use of tivantinib in patients with MiT family translocation RCCs pointed out neither survival benefits nor promising response rates related to this TKI [102].

**Table 2:** Tyrosine kinase inhibitors assessed in pRCC: recent published trials.

Trials	Histologies	Drug	Setting	N. of Patients	ORR (%)	mOS (Months)	mPFS (Months)
SWOG 1500/PAPMET (phase II) [92]	pRCC	-cabozantinib -sunitinib -savolitinib -crizotinib	First or second line	−44 −46 −29 −28	−23 −4 −3 −0	−20.4 −16.4 −11.7 −19.9	9.0 −5.6 −3.0 −2.8
SAVOIR (phase III) [96]	pRCC	-savolitinib -sunitinib	First or later line	−33 −27	−27 −7	-NR −13.2	−7.0 −5.6
SUPAP (phase II) [65]	pRCC: -type 1 -type 2	-sunitinib	First line	−15 −46	−13 −11	−17.8 −12.4	−6.6 −5.5
CREATE (phase II) [98]	pRCC: -type 1 -MET-driven type1 -MET-independent type1	-crizotinib	First or later line	−23 −4 −19	−17 −50 −11	−30.5 -NA −14.5	−5.8 -NA −3.0
SWOG S1107 (phase II) [100]	pRCC	-tivantinib -tivantinib + erlotinib	First or second line	−25 −25	−0 −0	−10.3 −11.3	−2.0 −3.9
NCT00726323 (phase II) [99]	pRCC	-foretinib	First or second line	74	13.5	NA	9.3
NCT02127710 (phase II)	-pRCC -MET-driven pRCC -MET-independent pRCC	-savolitinib	First or later line	−109 −44 −65	−7 −18 −0	-NA -NA -NA	-NA −6.2 −1.4

Abbreviations: pRCC = papillary renal cell carcinoma; NA = not available; NR = not reached; ORR = overall response rate; mPFS = median progression-free survival; mOS = median overall survival.

## 4. Immunotherapy and New Therapeutic Perspectives in nccRCC

As shown in several recent trials, RCC is a tumor with a mild to high percentage of somatic mutations, leading to an overexpression of neoantigens and then to a high immunogenic power. As a proof of it, renal cancer is characterized by a dysfunctional immune cell infiltrate along with an immune suppressive microenvironment [103,104,105]. Cancer-induced immune suppression is one of the most effective strategies that allows tumor growth. Notably, this tumoral “immune escape” is mostly mediated by programmed death protein-1 (PD-1) and cytotoxic T-lymphocyte associated antigen-4 (CTLA4), which are expressed on T cells’ plasmalemma and may trigger inhibitory pathways blocking the anti-cancer immune response, thus explaining the revolutionary use of anti-PD-1/PD-L1 and anti-CTLA-4 agents in several tumors. Thanks to more detailed studies, it was pointed out that many other strategies pursue the same results [89]. In 2017, Chen et al. described three basic immune profiles that might be related to patients’ response to anti-PD-L1/PD-1 therapy. Whereas the “immune desert” tumors are characterized by the total lack of intratumoral immune cell infiltration (because of immunological ignorance, induction of tolerance or absence of appropriate T-cell priming), the “immune excluded” tumors exploit many strategies to hide from immune response, such as the development of angiogenesis and extracellular matrix. Lastly, the “inflamed” tumors are characterized by a rich intratumoral infiltrate of CD8+ and CD4+ T-lymphocytes, along with myeloid and monocytic cells, that are restrained by the above-mentioned “immune escape” mechanisms. The latter profile may be the result of a pre-existing antitumor immune response that was arrested, probably by immunosuppression in the malignant microenvironment [106].

This “immune escape” strategy appears to be linked to no single gene alterations. Otherwise, the most plausible hypothesis is that this escape is due to different mutated genes which cooperate with each other. With regards to renal cancers, the Cancer Genome Atlas molecular characterization of RCCs carried out a comprehensive immune signature gene profiling demonstrating that, with the exception of Th17 (mostly characterizing chRCC), IL-8 and CD56^bright^NK (more expressed in pRCCs) cell genes, the others were more significantly overexpressed in ccRCC than pRCC and chRCC [107]. Furthermore, the Th2 gene signature (related to T-lymphocyte regulatory activity) was shown to be significantly upregulated in most of ccRCC, in all pRCC with a CpG island methylator phenotype (also known as CIMP-pRCC), and in some pRCC and chRCC; this signature may be considered a negative prognostic factor, regardless of tumor histologies [107]. The TCGA also noticed that ccRCC maximally expresses specific genes such as *PDCD1* and *CD247* (coding for PD1 and PD-L1), in comparison to pRCC and chRCC [107]. In addition, immune inflamed tumors were described to be lacking *PBRM1* alterations, being characterized by chromosomal losses of 9p21.3 instead. Whereas the *PBRM1* wild type seems to positively influence patients’ response to anti-PD-1 treatments, losses of 9p21.3 appear to be related to a worse outcome with PD-1 inhibitors. Nonetheless, tumor mutation burden, neoantigen overexpression, and HLA zygosity were not associated with higher anti-PD-1 therapy sensibility [108].

As stated before, the improvement in “immune escape” knowledge has led researchers to investigate novel treatment approaches for patients with advanced RCC using PD-1/PD-L1- and CTLA-4-targeted ICIs. While the immunotherapy has been assessed in ccRCC since 2015 [109], the first promising data about non-clear cell histologies have been recently highlighted in the 2021 phase II KEYNOTE 427 trial [110]. The KEYNOTE 427 trial investigated the efficacy and safety of the anti-PD-1 pembrolizumab as monotherapy for untreated patients with advanced ccRCC (cohort A) and advanced nccRCC (cohort B). Focusing on cohort B patients, first-line pembrolizumab showed promising clinical activity (ORR 26.7%) in the overall nccRCC population, regardless of International Metastatic RCC Database Consortium (IMDC) risk groups, and granted consistent results in selected patient subgroups with tumors with high PD-L1 expression [110]. When evaluated by RCC histology, response rates were higher for patients with papillary and unclassified RCC than for patients with chRCC [110]. Given the better survival traditionally related to the chromophobe histotype, further studies are necessary to explain this unclear finding.

Lee and colleagues have recently presented the primary results of the use of the nivolumab + cabozantinib combination in metastatic nccRCC patients, at the American Society of Clinical Oncology (ASCO) Symposium 2021 [111]. This phase II study (NCT03635892) tested the administration of nivolumab and cabozantinib in treatment-naïve, or previously treated with one VEGF-R TKI or mTOR inhibitor, patients with pRCC, MiT family translocation RCC, unclassified RCC (cohort 1), or chRCC (cohort 2). The above-mentioned immunocombination showed an acceptable safety profile along with a promising clinical efficacy in all the histologies included in the cohort 1. Considering a 13.1-month long median follow-up, cohort 1 patients witnessed a median PFS of 12.5 months (95% CI: 6.3–16.4) and a median OS of 28 months (95% CI: 16.3–NE). The primary endpoint ORR for cohort 1 was 48% (95% CI: 31.5–63.9). On the other hand, no response emerged in the chromophobe subtype (ORR for cohort 2 was 0%), confirming the poor clinical efficacy of ICI-based regimens in chRCC already highlighted in the KEYNOTE 427 trial.

Several trials are currently underway assessing the activity of ICIs in non-clear cell histotypes, such as UNISoN (NCT03177239) and SUNIFORECAST (NCT03075423), which are investigating the use of nivolumab with or without ipilimumab in nccRCC patients. Furthermore, promising preliminary data have recently been obtained with regards to the coadministration of bevacizumab and atezolizumab among nccRCC patients (NCT02724878) [112] or the combination of savolitinib and durvalumab in pRCC (NCT02819596, CALYPSO trial) [113]. The phase III CONTACT-03 trial is currently evaluating the efficacy of atezolizumab plus cabozantinib in patients with advanced nccRCC progressed after an ICI-based first-line therapy (NCT04338269). Promising preliminary results come from other ongoing studies testing cabozantinib in combination with atezolizumab (COSMIC-021, NCT03170960) or with nivolumab and ipilimumab (COSMIC-313, NCT03937219) [114,115]. Additionally, the phase II KEYNOTE-B61 trial is currently testing the coadministration of anti-PD-1 pembrolizumab with TKI lenvatinib as first-line strategy among metastatic nccRCC patients (NCT04704219) [116].

It must be considered noteworthy the significant ICI-based regimens’ antitumor activity in RCC with sarcomatoid and rhabdoid features (S/R RCC), whose response to classic RCC treatments (including anti-angiogenic agents as well as mTOR inhibitors) is poor [117]. As highlighted in the up-to-date work by Bakouny and colleagues, S/R RCCs harbor a significantly high rate of distinctive genomic alterations that lead to the development of an “inflamed” phenotype, thereby supporting their response to ICIs. A significant clinical benefit has been noticed for patients with S RCC treated with first-line ICI-based regimens, as pointed out in four large clinical trials in which these combinations were compared to sunitinib (CheckMate 214, JAVELIN Renal 101, IMmotion151, and KEYNOTE 426) [118,119,120,121]. Lastly, in an exploratory subgroup analysis of 75 patients with sarcomatoid features of the CheckMate 9ER trial, nivolumab + cabozantinib displayed a reduction of 64% of death risk compared to sunitinib (HR 0.36; 95% CI: 0.17–0.79), and an ORR of 55.9% vs. 22.0% [122]. In Table 3 we report the most relevant published and underway phase II/III trials, testing the clinical activity of ICI-based regimens in non-clear cell disease. In Table 4 we display the results of phase III pivotal studies assessing immuno-combinations in mRCC with regards to S RCC subgroups.

As emerged from these data, our knowledge about the interaction between RCC and the immune system is in constant development. Indeed, several novel strategies are emerging for the management of RCC and are in the course of investigation. Among these, strategies of certain interest include the use of CIK cells (a heterogeneous population of effector CD3+CD56+ NK T cells) combined with the anti-PD-1 drug camrelizumab (NCT03987698) and the chance to combine polyethylene glycolylated IL-2 (NKTR-214) to VB10.NEO, an individualized DNA plasmid cancer vaccine [123]. Furthermore, a new class of promising compounds, currently under investigation in multiple human cancers in the context of in vivo and in vitro experiments, are the SOD mimics. Mostly in RCC, the importance of oxidative stress has been widely demonstrated. MnP (MnTnHex-2-PyP5+), one of these molecules, has been studied in an in vitro experimentation performed on 786-O cells (a human cell line derived from primary clear-cell adenocarcinoma from a male patient). MnP showed a certain cytotoxic activity and the enhancement of intracellular ROS, suggesting its potential role in reducing the migration of renal cancer cells. Clearly, further studies about this and other SOD mimic compounds are needed to better analyze their potential function in treating clear cell and non-clear cell RCC [124].

## 5. Conclusions

nccRCC comprises a wide diversity of histologies with different molecular alterations and prognosis. The knowledge on the genetic features guiding the development and progression of the different subtypes is expanding through molecular based analysis thus leading to a widening of the therapeutic scenario.

Open issues that need to be solved are the validation of biomarkers for early prediction of RCC progression, monitoring and response to treatment [3]. An improvement in this field could also lead to a better tailored management.

In addition, another unmet clinical need is to define the best treatment sequence, taking into account the promising results of the PAPMET trial, CheckMate 9ER, and also considering what might emerge from the other ongoing studies [125]. In particular, a great response to nivolumab plus cabozantinib has been highlighted in sarcomatoid RCC, changing the practice of these poor prognosis tumors.

Despite these encouraging results, it is still hard to enroll a significant number of patients in large prospective randomized clinical trials (also because of the rarity of the histological subtypes of nccRCC), which are necessary to answer all these questions.

## Figures and Tables

**Table 1:** Histotypes categorized as non-clear cell renal cell carcinoma (nccRCC) along with their epidemiological features and genetic abnormalities.

Histotype	Frequency (% of All RCCs)	Cytogenetic Mutations	Genes Altered
Type 1 papillary RCC	10–15% (considering all pRCCs)	+3q, +7, +8q, +12q, + 16p, +17, +20, −9p, −Y	*MET*, *CUL3, NFR2, TERT,* *CDKN2A/B, EGFR*
Type 2 papillary RCC	10–15% (considering all pRCCs)	+7, +8q, +12, +16, +17, −1p, −9p, CpG island methylator phenotype, chromothripsis	*CDKN2A* silencing, *SETD2,* *NF2, CUL3, TERT* promoter, fumarate hydratase (*FH*)
Chromophobe RCC	5%	−1, −2, −6, −7, −10, −13, −17, −21	*TP53, PTEN,* hypodiploidy *KIT*
Oncocytoma	3–7%	Diploid karyotype, loss of chromosome 1 or Y, or rearrangement of 11q13.	Mitochondrial genes (*COX1, COX2, MTND4, MTCYB*). The 11q13 rearrangement may affect the *CCND1* gene.
Collecting duct carcinoma (also known as Bellini’s carcinoma)	1%	−1q, −8p, −9p, −16p, +13q	*NF2, SETD2, SMARCB1,* *FH, CDKN2A*
Medullary RCC	1%	*ABL/BCR* (rare), *SMARCB1/ALK* (rare), monosomy 11	Not defined
MiT family translocation RCC	1%	Recurrent translocations involving Xp11.2 (*TFE3*) or 6p21 (*TFEB*)	Not defined
Multilocular cystic renal neoplasm of low malignant potential	<1%	Not defined	Not defined
Hereditary leiomyomatosis with RCC	<1%	Not defined	*FH*
Succinate dehydrogenase-deficient RCC	<1%	Not defined	*SDH* (double hit inactivation)
Acquired cystic kidney disease-associated RCC	<1%	+3, +7, +17, −Y	Not defined
Unclassified RCC	about 5%	Not defined	Not defined

Abbreviations: RCC = renal cell carcinoma; pRCC = papillary renal cell carcinoma.

**Table 3:** A summary of the main published and ongoing phase II and III studies evaluating immunotherapies in non-clear cell histotypes.

Clinical Trial (phase)	Experimental Arm	Histology	Setting	Primary Endpoint
KEYNOTE 427 (phase II) [110]	Pembrolizumab	pRCC type 1 and 2, chRCC, unclassified nccRCC	Previously untreated metastatic ccRCC (cohort A) and nccRCC (cohort B)	ORR (26.7% in the overall nccRCC population)
NCT03635892 (phase II) [111]	Nivolumab + cabozantinib	pRCC type 1 and 2, chRCC, MiT family translocation RCC, unclassified nccRCC	Previously untreated or treated with a prior VEGF-R TKI/mTORi metastatic pRCC, MiT family translocation RCC, unclassified RCC (cohort 1) and chRCC (cohort 2)	ORR (48% in cohort 1, 0% in cohort 2)
CONTACT-03 (phase III)	Atezolizumab + cabozantinib	All non-clear cell subtypes	Locally advanced or metastatic RCC in PD during or after one ICI-based regimen	PFS and OS (no results posted, recruiting underway)
COSMIC-021 (phase Ib/II) [114]	Atezolizumab + cabozantinib	All non-clear cell subtypes	Previously untreated locally advanced, metastatic, or recurrent solid tumors (including ccRCC and nccRCC)	ORR (no results posted)
COSMIC-313 (phase III) [115]	Nivolumab + ipilimumab + cabozantinib	All non-clear cell subtypes	Previously untreated IMDC intermediate-/poor-risk metastatic RCC (including ccRCC and nccRCC)	PFS (no results posted)
UNISoN (phase II)	Nivolumab (for a maximum of 12 months), then nivolumab + ipilimumab (4 cycles) and lastly maintenance with nivolumab single agent	pRCC type 1 and type 2, chRCC, S RCC, Xp11 translocation RCC, unclassified nccRCC	Metastatic nccRCC previously untreated or treated with a VEGF-R TKI or another systemic therapy	ORR (no results posted)
SUNIFORECAST (phase II)	Nivolumab + ipilimumab	All non-clear cell subtypes	Previously untreated locally advanced or metastatic nccRCC	OS (no results posted)
CALYPSO (phase Ib/II) [113]	Savolitinib + durvalumab	pRCC type 1 and type 2	Previously untreated metastatic ccRCC (cohort A) and nccRCC (cohort B)	DLT, ORR (no results posted)
NCT02724878 (phase II) [112]	Atezolizumab + bevacizumab	All non-clear cell subtypes	Previously untreated locally advanced or metastatic nccRCC	ORR (33% in the experimental group)
KEYNOTE-B61 (phase II) [116]	Pembrolizumab + lenvatinib	All non-clear cell subtypes	Previously untreated locally advanced or metastatic nccRCC	ORR (no results posted, recruiting underway)

Abbreviations: pRCC = papillary renal cell carcinoma; chRCC = chromophobe renal cell carcinoma; nccRCC = non-clear cell renal cell carcinoma; ccRCC = clear cell renal cell carcinoma; S RCC = renal cell carcinoma with sarcomatoid features; ORR = overall response rate; PFS = progression-free survival; OS = overall survival; DLT = dose limiting toxicity; PD = disease progression; ICI = immune checkpoint inhibitor; VEGF-R TKI = vascular endothelial growth factor receptor’s tyrosine kinase inhibitor; mTORi = mTOR inhibitor; IMDC = International Metastatic RCC Database Consortium.

**Table 4:** Survival outcomes and antitumor activity of ICI-based regimens among RCC with sarcomatoid features.

	CheckMate 9ER [122]	KEYNOTE 426 [121]	Javelin RENAL 101 [119]	CheckMate 214 [118]
Experimental arm	Nivolumab + cabozantinib	Pembrolizumab + axitinib	Avelumab + axitinib	Nivolumab + ipilimumab
mOS (months)	NR (95% CI, 22.8–NE) HR 0.36 (95% CI, 0.17–0.79)	NR HR 0.58 (95% CI, 0.21–1.59)	Data unavailable	NR (95% CI, 25.2–NE) HR 0.45 (95% CI, 0.30–0.70)
mPFS (months)	10.3 (95% CI, 5.6–19.4) HR 0.42 (95% CI, 0.23–0.74)	NR HR 0.54 (95% CI, 0.29–1.00)	7.0 (95% CI, 5.3–13.8) HR 0.57 (95% CI, 0.325–1.003)	26.5 HR 0.54 (95% CI, 0.33–0.86)
ORR (CR)	55.9% (CR unavailable)	58.8% (13%)	46.8% (4.3%)	60.8% (18.9%)

Abbreviations: ORR = overall response rate; CR = complete response rate; mPFS = median progression-free survival; mOS = median overall survival; ICI = immune checkpoint inhibitor; NR = not reached; NE = not estimable.

## Data Availability

Not applicable.
